# Evaluation of Functionalized Amberlite Type XAD7 Polymeric Resin with L-Valine Amino Acid Performance for Gallium Recovery

**DOI:** 10.3390/polym16060837

**Published:** 2024-03-18

**Authors:** Cosmin Vancea, Loredana Ciocarlie, Adina Negrea, Giannin Mosoarca, Mihaela Ciopec, Narcis Duteanu, Petru Negrea, Bogdan Pascu, Nicoleta-Sorina Nemes

**Affiliations:** 1 Faculty of Industrial Chemistry and Environmental Engineering, Politehnica University Timisoara, Bd. V. Parvan No. 6, 300223 Timisoara, Romania; cosmin.vancea@upt.ro (C.V.); loredana.ciocarlie@student.upt.ro (L.C.); mihaela.ciopec@upt.ro (M.C.); narcis.duteanu@upt.ro (N.D.); petru.negrea@upt.ro (P.N.); 2 Renewable Energy Research Institute-ICER, Politehnica University Timisoara, Gavril Musicescu Street No. 138, 300501 Timisoara, Romania; ioan.pascu@upt.ro (B.P.); nicoleta.nemes@upt.ro (N.-S.N.)

**Keywords:** gallium recovery, adsorption, DL-valine amino acid, Amberlite XAD7

## Abstract

Given the ever-increasing demand for gallium(III) as a crucial precursor in the fabrication of advanced materials, there arises an imperative to devise efficient recovery processes from primary and secondary sources. In the present investigation, the retrieval of gallium(III) from aqueous solutions through the mechanism of adsorption was investigated. Materials with superior adsorbent properties play an important role in the dynamics of the adsorption process. To enhance these properties, select materials, such as Amberlite-type polymeric resins, are amenable to functionalization through impregnation with extractants featuring specialized active groups, designed for the selective recovery of metal ions—specifically, Ga(III). The impregnation method employed in this study is the Solvent-Impregnated Resin (SIR) method, utilizing the amino acid DL-valine as the extractant. The new material was characterized through Scanning Electron Microscopy (SEM), Elemental Analysis via X-ray energy-dispersive spectroscopy (EDX), and Fourier transform infrared spectroscopy (FTIR) to elucidate the presence of the extractant on the resin’s surface. Concurrently, the material’s pH_PZC_ was determined. The adsorptive prowess of the synthesized material was investigated through kinetic, thermodynamic, and equilibrium studies. The influence of specific parameters in the adsorption process—namely, pH, contact time, temperature, and Ga(III) initial concentration—on the maximal adsorption capacity was determined. The optimal adsorption conditions were established using the Taguchi method.

## 1. Introduction

Gallium (Ga) was discovered in 1875 [1]. Its presence in the Earth’s crust is commensurate with metals like cobalt, lead, and niobium. Gallium primarily manifests as a sulfide or hydrated oxide, ubiquitously distributed as an impurity within diverse minerals. The oxides and sulfides of gallium exhibit low solubility in water, contributing to its sparse concentration in seas and rivers. Notably, the gallium-rich mineral gallite (CuGaS_2_) is the sole source; however, practical challenges preclude its extraction on a significant scale. Another lesser-known gallium-containing mineral is gallium plumbogummite. Naturally, there are no ores with an ideal gallium concentration surpassing 0.1% by mass. Rather, gallium is acquired through the secondary process of the metallurgical treatment of ores containing different metals. Examples include extraction from bauxite, zinc blende, coals, galenas, pyrites, germanites (wherein gallium coexists with aluminum), zinc, carbon, lead, iron, and germanium [2].

This metallic element is important for its applications in various domains [3]. The industrial utilization of gallium(III) experienced a pronounced upswing starting in the 1970s, concomitant with the revelation of the semiconducting properties exhibited by gallium(III) compounds, particularly gallium arsenide (GaAs) and gallium nitride (GaN), in conjunction with group 15 elements [4]. These advanced materials find widespread application in the electronics field, in devices such as mobile phones, photovoltaic generation panels, optical communication devices, and computers. Notably, GaAs and GaN are important components in the fabrication of integrated circuits and mobile telecommunications, and exhibit utility in military applications. Beyond electronics, Ga(III) assumes an essential role as a constituent in diverse alloys characterized by low melting points, including plutonium alloys with specific applications within the nuclear weapons industry [5,6].

Gallium has also replaced mercury in thermometry, serving as a liquid medium to gauge temperatures indicated by thermometers. However, its melting point of 29.7 °C proves somewhat too elevated for this application. Consequently, in its metallic state, its direct use in thermometers is deemed impractical. Instead, an alloy named Galinstan (Ga-In-Sn), featuring a lower melting point, is used for this purpose [7]. Galinstan alloy has a melting point of approximately −18 °C and its lack of toxicity makes it an ideal substance for the design of mercury-independent medical thermometers. This feature renders it a convenient material, ensuring safe cleanup in the event of breakage, albeit with the caveat of surface wetting, leading to potential floor soiling [7].

Beyond its thermometric applications, gallium alloys have found utility in the creation of diverse objects, facilitated by their ability to solidify upon cooling. This property unveils promising nanotechnological prospects for fabricating minute structures capable of exhibiting unique gallium-based properties at lower temperatures [8].

Gallium oxides have been used for catalytic studies in various organic reactions of notable industrial relevance. A recent advancement involves a gallium-based catalyst, comprising liquid gallium as a matrix, wherein dispersed atoms of other metals serve as active centers or catalytic sites [9]. For instance, a gallium–palladium catalyst was used in the butane dehydrogenation reaction, involving the transformation of butane into more reactive unsaturated species essential for other industrial processes [10].

Efficient techniques for the recovery of gallium(III) from its sources have attracted significant scientific interest. However, the translation of these techniques into practical industrial methods remains limited. Despite some existing literature reviews on Ga(III) recovery [4,5,11], there is a dearth of information concerning Ga(III) resources and recovery methodologies, particularly given the rapid evolution of the Ga(III) industry in recent years [12].

Various methodologies have been devised for Ga(III) recovery from aqueous solutions, encompassing electrochemical processes such as mercury cathode electrolysis [13], cementation [14,15,16,17,18], precipitation, solvent extraction, ion exchange [19], and adsorption techniques [20,21,22,23,24].

This study aims to recover Ga(III) from aqueous solutions through adsorption onto a novel material derived from the functionalization of a commercial Amberlite XAD7-type polymeric resin via impregnation with the amino acid DL-Valine. The employed functionalization method, namely the Solvent-Impregnated Resin (SIR) method, is designed to enhance the adsorbent properties of the inert support, i.e., the XAD7 resin. DL-Valine, selected as the extractant, stands out due to its environmental friendliness, its cost-effectiveness, and the presence of active functional groups (-NH_2_ and -COOH) within its molecular structure, which augment the adsorptive capabilities of the material.

## 2. Materials and Methods

### 2.1. Characterization and Preparation of Adsorbents

#### 2.1.1. Functionalization of Amberlite XAD 7 Resin with DL-Valine

The commercial polymer resin employed in this study is Amberlite XAD7 (Sigma-Aldrich, St. Louis, MO, USA), characterized by an acrylic matrix featuring particles within the 20–60 mesh size range, a pore volume of 0.5 mL/g, an average pore diameter of 300 Å, and a surface area of 380 m^2^/g. The functionalization process involved impregnating this resin with the amino acid DL-valine (2-amino-3-methylbutanoic acid-C_5_H_11_NO_2_) acquired from Sigma-Aldrich (St. Louis, MO, USA). The chosen impregnation method was the dry Solvent-Impregnated Resin (SIR) method. The mass ratio of the support (resin) to extractant (DL-valine) was maintained at 10:1. The support and extractant, once dissolved in water, underwent a 24 h contact period, followed by drying in an oven (Pol-eko model SLW 53, Wodzisław Śląski, Poland) at 323 K for an additional 24 h.

#### 2.1.2. Characterization of XAD7-Va Material

The characterization of the adsorbent is important for its application in the adsorption processes. Thus, in order to highlight the presence of the amino acid DL-valine on the surface of the resin Amberlite XAD 7, the synthesized material was characterized by scanning electron microscopy (SEM) and by elemental analysis via X-ray energy-dispersive spectroscopy (EDX) using a Quanta FEG 250 instrument (FEI, Eindhoven, The Netherlands). Also, the functional groups specific to the support, but especially the extractant, were highlighted by Fourier transform infrared spectroscopy using an IRAffiniy-1S SHIMAZDU spectrophotometer (Shimadzu, Kyoto, Japan).

The point of zero charge, the pH_PZC_, was also determined using the method of bringing the studied system to equilibrium. All reagents involved in the analysis were purchased from Sigma-Aldrich (St. Louis, MO, USA). For this study, a quantity of 0.1 g of material, XAD7-Va, was used, which was mixed with 25 mL of KCl 0.1 N solution. The mixture was agitated at a speed of 200 rotations per minute and maintained at a temperature of 298 Kelvin using a water bath equipped with a thermostat and Julabo SW23 shaker apparatus (Julabo GmbH, Seelbach, Germany). The acidity of the KCl solutions was balanced within a pH range of 2–12 by using NaOH solutions ranging in concentration from 0.05 N to 2 N or HNO_3_ solutions ranging in concentration from 0.05 N to 2 N. The samples were then filtered before measuring the pH of the resulting solution using a METTLER TOLEDO pH-meter (Mettler Toledo, Columbus, OH, USA).

### 2.2. Effect of Adsorption Parameters on Ga(III) Recovery

#### 2.2.1. pH Effect

In this research, the impact of pH variations on the adsorption kinetics of Gallium(III) (as Ga(NO_3_)_3_ in HNO_3_ 2–3%, at a concentration of 1000 mg/L Gallium, Certipur from Merck KGaA, Darmstadt, Germany) onto the adsorbent material was investigated. The pH of the solution was adjusted within the range of 1–10. The experimental conditions included an initial Ga(III) concentration (C_0_) of 10 mg/L, employing 0.1 g of the adsorbent material, a solution volume of 25 mL, a contact time of 60 min, and a temperature of 298 K.

#### 2.2.2. Contact Time and Temperature Effect: Kinetic and Thermodynamic Studies

The impacts of contact time and temperature are crucial factors in assessing the attraction of Ga(III) to the material. In order to determine the influence of contact time and temperature on the adsorption capacity of the synthesized XAD7-Va material, 0.1 g of the material was weighed, and 25 mL of a Ga(III) solution with a concentration of 10 mg/L was introduced. The samples were stirred for varying durations (15, 30, 45, 60, 90, and 120 min) within a controlled water bath environment, featuring precise temperature regulation and stirring capabilities. Distinct temperatures (298 K, 308 K, 318 K, and 328 K) were used during the experimental process. The stirring occurred at a rate of 200 rpm, maintaining a working pH above 3.

Subsequent to the adsorption process, the residual concentration of Ga(III) was quantified utilizing atomic adsorption spectrometry.

The kinetic models used in the study of Ga(III) adsorption on the XAD7-Va material, known from the literature, are presented below along with their non-linear equations:

Pseudo-first-order:

(1) $$q_{t}=q_{e}\left(1-{exp}^{-k_{1}\cdot t}\right)$$

Pseudo-second-order:

(2) $$q_{t}=\frac{k_{2}\cdot t\cdot q_{e}^{2}}{1+k_{2}\cdot {t\cdot q}_{e}}$$

Elovich:

(3) $$q_{t}=\frac{1}{a}ln\left(1+a\cdot b\cdot t\right)$$

Avrami:

(4) $$q_{t}=q_{AV}\left[1-exp{\left(-k_{AV}\cdot t\right)}^{n_{AV}}\right]$$

where *q_t_* is the amount adsorbed at time *t*; *k*_1_, *k*_2_, and *k_AV_* are the rate constants of the pseudo-first-order, pseudo-second-order, and Avrami models; *q_e_*, and *q_AV_* are theoretical values for the adsorption capacity; *a* is the desorption constant of the Elovich model; *b* is the initial velocity; and *n_AV_* is the fractional exponent [25,26].

To establish whether the adsorption process of Ga(III) on the surface of the XAD7-Va material proceeds spontaneously, the value of Gibbs free energy can be calculated using the Gibbs–Helmholtz equation [27]:

(5) $$\Delta G^{0}=\Delta H^{0}-T\cdot \Delta S^{0}$$

where Δ*G*^0^ is the standard variation of Gibbs free energy (kJ/mol); Δ*H*^0^ is the standard variation of enthalpy, (kJ/mol); Δ*S*^0^ is the standard variation of entropy, (J/mol·K); and *T* is the absolute temperature, (K).

Using the van’t Hoff equation, the standard variation of entropy Δ*S*^0^ and the standard variation of enthalpy Δ*H*^0^ can be calculated from the equation of the line obtained from the graphical representation of ln K_d_ as a function of 1/*T*:

(6) $$\ln\;K_{d}=\frac{\Delta S^{0}}{R}-\frac{\Delta H^{0}}{R\cdot T}$$

where *K_d_* is the equilibrium constant; Δ*S*^0^ is the standard variation of entropy, (J/mol·K); Δ*H*^0^ is the standard variation of enthalpy, (kJ/mol); *T* is the absolute temperature, (K); and *R* is the ideal gas constant, (8.314 J/mol·K).

The equilibrium constant is the ratio between the equilibrium adsorption capacity, *q_e_*, and the equilibrium concentration, *C_e_*:

(7) $$K_{d}=\frac{q_{e}}{C_{e}}$$

where *q_e_* is the equilibrium adsorption capacity (mg/g), and *C_e_* is the equilibrium concentration (mg/L).

#### 2.2.3. Ga(III) Initial Concentration Effect: Equilibrium Studies

For the equilibrium studies, 0.1 g of XAD7-Va material was weighed, over which 25 mL of samples with different Ga(III) concentrations (0.1, 0.3, 0.5, 1, 3, 5, 10, 20, 40, 60, 80, 100, and 110 mg/L). The adsorption studies were carried out for 1 h at 298 K, pH > 3, using a water bath with a thermostat and stirring (200 rpm). After the contact time expired, the samples were filtered, and then, the residual Ga(III) concentration in the solution was determined by atomic absorption spectrometry using an atomic absorption spectrophotometer.

The acquisition of equilibrium data, commonly referred to as adsorption isotherms, serves as a fundamental prerequisite to understanding the mechanism of the adsorption process. Several isotherms were employed in this study, each characterized by non-linear equations as delineated below:

Langmuir:

(8) $$q_{e}=\frac{q_{m}\cdot K_{L}\cdot C_{e}}{1+K_{L}\cdot C_{e}}$$

Freundlich:

(9) $$q_{e}=K_{F}\cdot C_{e}^{1/n_{F}}$$

Temkin:

(10) $$q_{e}=\frac{R\cdot T}{b}ln\left(K_{T}\cdot C_{e}\right)$$

Sips:

(11) $$q_{e}=\frac{Q_{sat}\cdot K_{S}\cdot C_{e}^{n}}{1+K_{S}\cdot C_{e}^{n}}$$

where *q_m_* and *Q_sat_* are the maximum absorption capacities; *K_L_*, *K_F_*, *K_T_* and *K_S_* are the Langmuir, Freundlich, Temkin, and Sips isotherms constants; 1/*n_F_* is the empirical constant indicating the intensity of adsorption; *R* is the universal gas constant; *T* is the absolute temperature; *b* is the Temkin constant, which is related to the adsorption heat; and *n* is the Sips isotherm exponent [28,29,30].

#### 2.2.4. Identifying the Most Appropriate Isotherm and Kinetic Models

Values for the determination coefficient (*R*^2^), the adjusted determination coefficient (*R*^2^*_adj_*), and chi-square (*χ*^2^) were computed to identify the most appropriate isotherm and kinetic models [25]. The suitability of these models was assessed by aiming for higher *R*^2^ and *R*^2^*_adj_* values, as well as a lower *χ*^2^ value. The equations for calculating these error parameters can be found below:

Determination coefficient (*R*^2^):

(12) $$R^{2}=1-\frac{\sum_{i=1}^{n}{\left(y_{i,exp}-y_{i,mod}\right)}^{2}}{\sum_{i=1}^{n}{\left(y_{i,mod}-\bar{y_{i,exp}}\right)}^{2}}$$

Adjusted determination coefficient (*R*^2^*_adj_*):

(13) $$R_{adj}^{2}=1-\left(\frac{n-1}{1-\left(n_{p}+1\right)}\right)\left(1+R^{2}\right)$$

Chi-square (*χ*^2^):

(14) $$\chi^{2}=\sum_{i=1}^{n}\frac{{\left(y_{i,exp}-y_{i,mod}\right)}^{2}}{y_{i,mod}}$$

where *y*_*i*,*exp*_ is the experimental value; *y*_*i*,*mod*_ is the modeled value; $\bar{y_{i,exp}}$ represents the mean values; *n* is the total amount of information; and *n_p_* is the model parameter number [25].

### 2.3. Optimization Using the Taguchi Method

In order to improve the effectiveness of removing Ga(III) ions, the Taguchi method was utilized. To find the best adsorption conditions, an experiment was conducted using an L_16_ orthogonal array, which involved four factors at four levels. The impact of each factor on the efficiency of Ga(III) removal was determined by performing an analysis of variance (ANOVA) with a general linear model. The necessary mathematical calculations were performed using Minitab 19 software (version 19.1.1, Minitab LLC, State College, PA, USA).

## 3. Results and Discussion

### 3.1. Characterization of the Adsorbents

#### 3.1.1. Scanning Electron Microscopy, SEM

**Figure 1:**
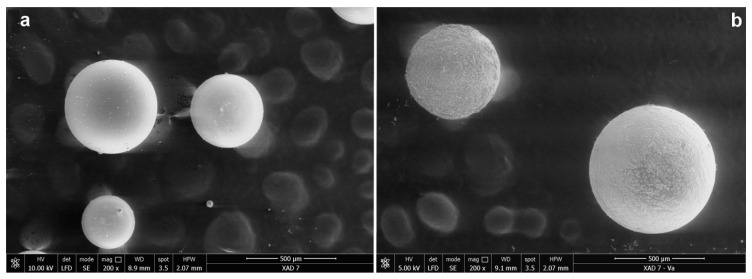
Scanning electron microscopy (SEM) for material before (**a**) and after functionalization (**b**).

Figure 1 illustrates scanning electron microscopy (SEM) micrographs for the Amberlite XAD7 polymer support, both antecedent to functionalization by impregnation (Figure 1a) and subsequent to the process (Figure 1b). A discernible contrast in the morphology of the resin spheres is highlighted in Figure 1. In particular, Figure 1b reveals the presence of white dots on the surface of the spheres, indicative of the amino acid DL-valine.

#### 3.1.2. X-ray Energy-Dispersive Spectroscopy, EDX

**Figure 2:**
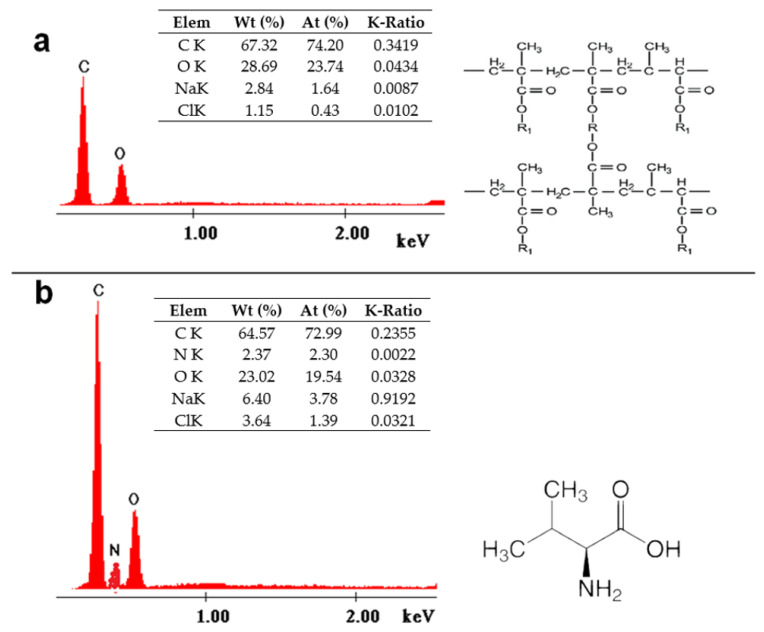
X-ray energy-dispersive spectroscopy for material before (**a**) and after functionalization (**b**).

Energy dispersive X-ray spectroscopy (EDX) facilitates elemental chemical analysis, offering insights into the composition of the studied materials. The EDX analysis data are illustrated in Figure 2. Notably, in the case of the XAD 7 resin functionalized with the amino acid DL-valine (Figure 2b), the presence of a specific nitrogen peak within the structure of the amino acid DL-valine is evident, confirming the successful functionalization of the surface of the XAD7 support, as corroborated by the absence of this peak in the EDX spectrum of the unmodified XAD7 resin (Figure 2a).

The mass and atomic percentages decrease in the case of the polymer functionalized with the amino acid Valine. At the same time, the appearance of 2.37% (W_t_) or 2.30% (A_t_) for N is noticed, which is specific to the -NH_2_ group in the amino acid structure.

#### 3.1.3. Fourier Transform Infrared Spectroscopy, FTIR

**Figure 3:**
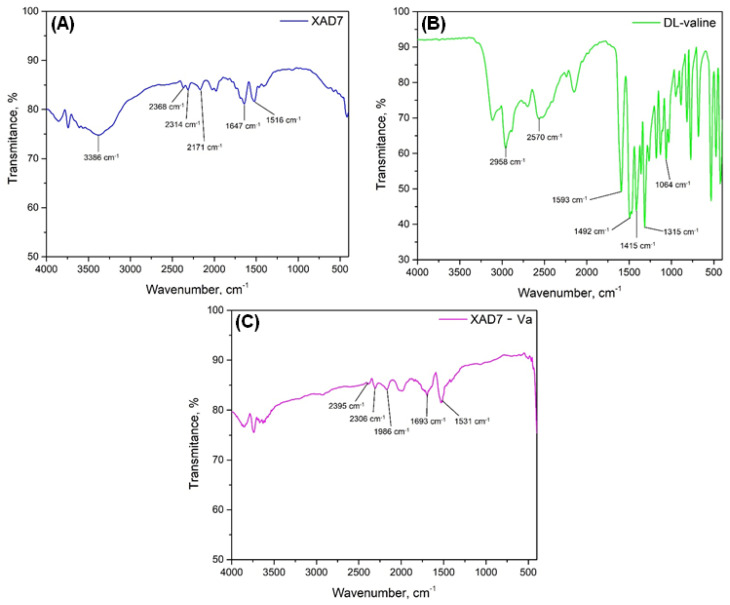
FTIR spectra: (**A**) Amberlite XAD 7 resin, (**B**) DL-Valine amino acid, (**C**) XAD7-Va material.

Figure 3 shows the FTIR spectra of Amberlite XAD7 resin (Figure 3A), the amino acid DL-valine (Figure 3B), and the material obtained after functionalization by impregnation (Figure 3C).

Specific to the Amberlite XAD7-type polymer support, the Fourier transform infrared (FTIR) spectrum reveals distinctive vibrational signatures. The spectrum manifests vibrations associated with the O–H bond at a wavenumber of 3375 cm^−1^, vibrations of the C=O bond at 1643 cm^−1^, and vibrations linked to the C–H bond deformations, including those specific to the -CH_3_ group at wavenumber 1519 cm^−1^ and those corresponding to the stretching of the C–H aliphatic group at wavenumbers 2364 cm^−1^, 2314 cm^−1^, and 2167 cm^−1^ [31].

Since the amount of the extractant employed in the functionalization process of DL-valine is very low (Amberlite XAD7/DL-valine ratio = 10:1), the specific peaks attributable to the amino acid are notably diminutive. Consequently, in the spectral region around 1531 cm^−1^, faint vibrations characteristic of the NH_2_ group or NH^3+^ symmetric deformation emerge, together with a low-intensity peak at 1300 cm^−1^ corresponding to specific COO- asymmetric stretching vibrations [32].

Adsorption studies were performed on non-functionalized Amberlite XAD7, and the adsorption capacity was insignificant, so the presence of specific valine groups on the surface of Amberlite XAD7, which are observed following XAD7 functionalization, significantly improve the adsorption/recovery process of Ga(III). The -NH_2_ and -COOH groups are very important in the adsorption mechanism of the Ga(III) ionic species present in aqueous media (Ga(OH)_2_^+^, Ga(OH)_3,_ sau Ga(OH)_4_^−^).

#### 3.1.4. Point Zero Charge, pH_PZC_

**Figure 4:**
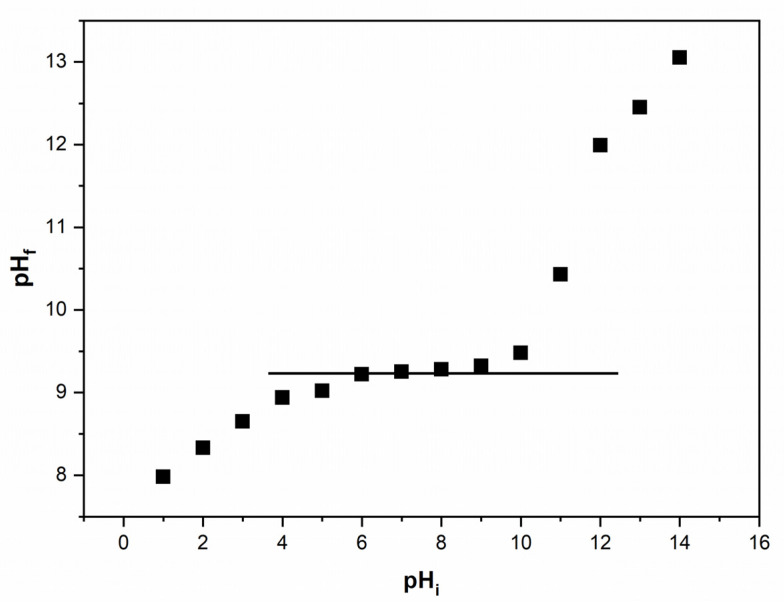
Determination of pH_PZC_ for XAD7-Va material.

The study of the point of zero charge (pH_PZC_) offers valuable information on the electric charge characteristics of the material surfaces. Figure 4 illustrates the pH_f_ versus pH_i_ dependency for the XAD7-Va material.

The point of zero charge (pH_PZC_) for the materials is approximately 9.25, indicating the pH at which the electric charge on the material surface is neutral. Consequently, at pH values greater than 9.25, the material surface carries a negative charge, exhibiting an affinity for cations. Conversely, at pH values lower than 9.25, the material surface is positively charged, demonstrating an affinity for anions.

### 3.2. Effect of Gallium Recovery Parameters on Adsorption Process

For each adsorption experiment, three independent replicates were performed, maintaining a constant stirring intensity. Each value presented represents an average of the three experimentally obtained values.

#### 3.2.1. pH Effect

**Figure 5:**
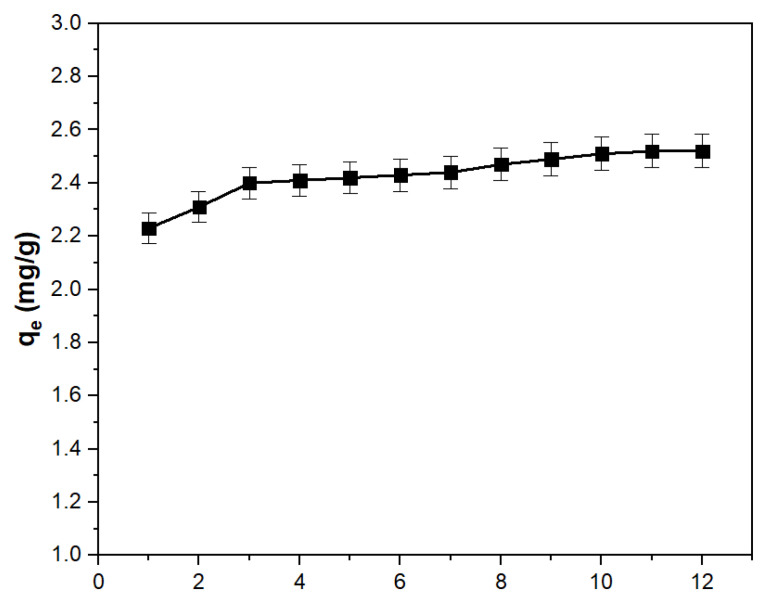
The effect of pH on the adsorption capacity.

The influence of the pH of the Ga(III) solution on the adsorption capacity is presented in Figure 5. It is discerned that the adsorption capacity exhibits a very slight increase, reaching the maximum value at a pH beyond the point corresponding to the point of zero charge. In accordance with existing literature data [33], the specific Ga(III) species coexisting in solution can be Ga(OH)_2_^+^, Ga(OH)_3_, or Ga(OH)_4_^−^.

#### 3.2.2. Contact Time and Temperature Effects: Kinetic and Thermodynamic Adsorption Studies

**Figure 6:**
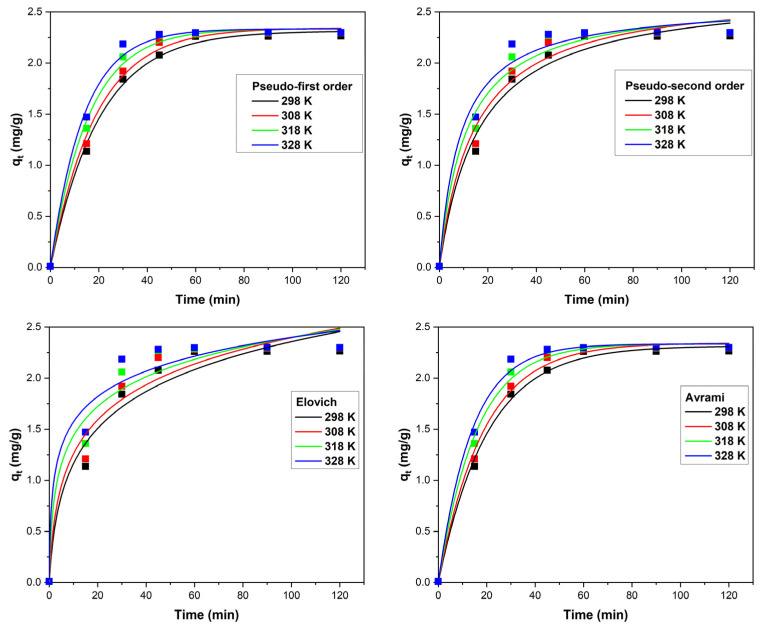
Contact time and temperature effects on the adsorption capacity together with the specific curves of the tested kinetic models.

In adsorption processes, a comprehensive understanding of the equilibrium dynamics necessitates the determination of contact time and the temperature at which the adsorbent–adsorbed system attains equilibrium. Illustrated in Figure 6 is the dependency of Ga(III) adsorption capacity on the XAD7–Va material concerning contact time at varying temperatures: 298 K, 308 K, 318 K, and 328 K.

The analysis of the experimental data reveals that the adsorption capacity of the XAD7-Va material progressively increases with extended contact time, reaching the maximum after 60 min. Consequently, this selected contact time of 60 min was considered optimal for subsequent investigations. The rise in temperature from 298 K to 328 K impacts the adsorption process; however, the minimal effect does not support the economic rationale for conducting studies at temperatures above 298 K.

To assess the kinetic mechanism governing Ga(III) adsorption onto XAD7-Va, the experimental data were subjected to modeling using nonlinear equations of the pseudo-first-order, pseudo-second-order, Elovich, and Avrami kinetic models. The curves derived from these models are illustrated in Figure 6, and the corresponding kinetic parameters for the four temperatures are presented in Table 1.

Analyzing the data within Table 1 highlights that both the kinetic pseudo-first-order model and Avrami models very accurately describe the adsorption process. Nevertheless, the most fitting model proves to be the pseudo-first-order model, substantiated by its superior values for *R*^2^ and *R*^2^*adj*, coupled with the lowest *χ*^2^ values.

**Figure 7:**
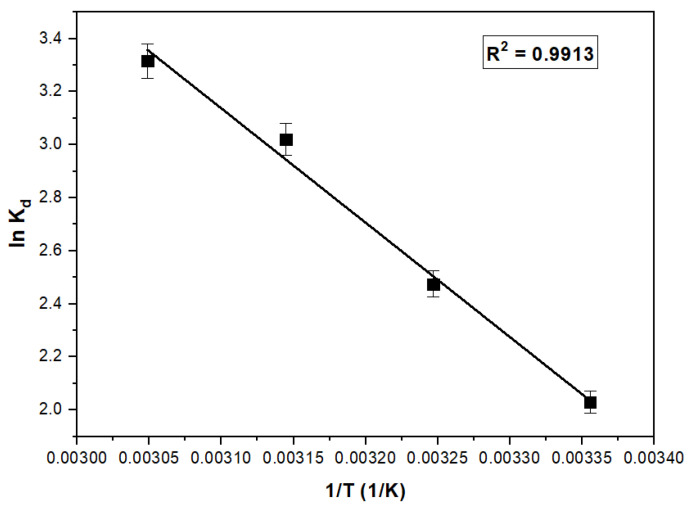
Plot of ln *K_d_* vs. 1/*T* for the estimation of thermodynamic parameters for the recovery Ga(III) on XAD7-Va.

To elucidate the impact of temperature on the adsorption capacity of Ga(III), a linearized representation of the experimental data is showcased in Figure 7.

Based on the acquired data, the thermodynamic parameters Gibbs free energy (Δ*G*^0^), enthalpy (Δ*H*^0^), and entropy (Δ*S*^0^) were calculated; their respective values are presented in Table 2. The positive enthalpy value, Δ*H*^0^, signifies that the energy required for the adsorption process is the energy used to bring Ga(III) ions into contact with the surface of the adsorbent material.

The standard Gibbs free energy change (Δ*G*^0^) exhibits a negative value and demonstrates a dependence on temperature, thereby signifying the spontaneity and thermodynamic favorability of Ga(III) adsorption on XAD7-Va. Concomitantly, the positive values of the standard enthalpy change (Δ*H*^0^) and standard entropy change (Δ*S*^0^) suggest the endothermic nature of the adsorption process and the heightened disorderliness observed at the solid–liquid interface.

The Δ*G*^0^ parameter ranges between −80 and −20 (kJ mol^−1^), suggesting a complex adsorption mechanism based on both physisorption and chemosorption [34,35].

#### 3.2.3. Adsorption Equilibrium

**Figure 8:**
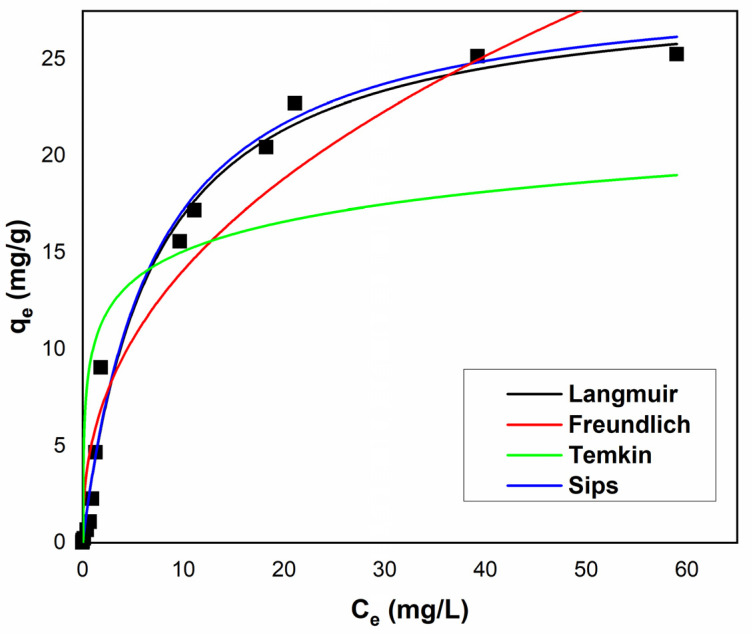
Isotherm model for adsorption of Ga(III) onto XAD7-Va.

To discern the behavior of Ga(III) ions on the surface of the XAD7-Va material during the adsorption process, the experimentally modeled data were subjected to analysis using four distinct equilibrium isotherms: Langmuir, Freundlich, Temkin, and Sips. The adsorption isotherms depicting Ga(III) on the polymeric material XAD7-Va are presented in Figure 8, with the corresponding isotherm parameters provided in Table 3.

Notably, the Langmuir isotherm manifests the highest values for the parameters *R*^2^ and *R*^2^*_adj_*, concurrently exhibiting the lowest value for the parameter *χ*^2^. These values indicate that the Langmuir isotherm best describes the experimental data obtained for the recovery of Ga(III) upon its adsorption on the XAD7-Va material, indicating a maximum value of adsorption capacity of 28.86 (mg/g).

In Table 4, the adsorption capacities for various synthesized or functionally modified materials employed for the removal of Ga(III) from aqueous solutions are presented comparatively. The efficacy of Amberlite XAD-type resins, particularly those functionalized with pendant groups, in the adsorption-driven removal of metal ions from aqueous solutions is well-established, as corroborated by the presented data. This conclusion is further reinforced by the comparative analysis with data extracted from the literature on the utilization of alternative materials for similar purposes.

### 3.3. Optimization Using Taguchi Method

Table 5 displays the four factors along with their respective levels, which served as the basis for the L_16_ orthogonal array experimental design. By implementing the Taguchi method, the most favorable adsorption conditions were determined.

The central aim of the Taguchi method is to obtain and analyze the signal-to-noise ratio (S/N) in order to evaluate the experiment’s quality and the validity of the results. The S/N ratio acts as a measure of the variability and accuracy levels for each response obtained in every trial. The signal represents the response obtained by manipulating each operational factor, while the noise refers to any factor that impacts precision. These elements are linked to the significance of the operational variable. The key benefits offered by this technique are the reduction of the number of experiments and the ability to visualize the optimal conditions. In order to enhance the efficiency of Ga(III) removal, the “larger is the better” choice was considered for the S/N ratio. [39,40,41]. The experimental results and the corresponding S/N ratios for each trial are shown in Table 6.

The signal-to-noise ratio for each level and the significance ranks of the factors (shown in Table 7) indicated that initial concentration had the greatest impact on the efficiency of Ga(III) removal, whereas temperature had the least effect. The Taguchi approach determined the optimal conditions for adsorption to be a pH of 10, an initial concentration of 60 (mg/L), a contact time of 90 (min), and a temperature of 318 K.

Table 7 also displays the results of the ANOVA analysis, indicating the percentage contribution of each controllable factor on Ga(III) removal efficiency. The values suggest that the order of the controllable factors’ influence matches that of the Taguchi method.

## 4. Conclusions

In this study, a novel material was synthesized through the chemical modification of the surface of a commercial Amberlite XAD7 polymer via functionalization through impregnation with the environmentally friendly amino acid DL-valine. The incorporation of active amino acid groups onto the polymer surface was elucidated through characterization techniques such as EDX, SEM, and FTIR. Additionally, the point of zero charge (pH_PZC_) of the material was determined.

The adsorption process of Ga(III) ions onto the XAD7-Va material was found to be spontaneous and endothermic, involving both physical and chemical adsorption mechanism at the adsorbent–adsorbate interface.

The modeling results suggest that the pseudo-first-order kinetic model is the best fitting model, while the Langmuir isotherm was identified as the most fitting representation of the adsorption process, showcasing a maximum adsorption capacity of 28.88 (mg/g).

The Taguchi method highlighted the initial concentration as being the primary influential factor, contributing significantly (66.88%) to the efficiency of Ga(III) removal, with temperature exerting a comparatively minimal effect (3.63%).

In conclusion, the synthesized XAD7-Va material, easy to synthesize, demonstrates good maximum adsorption capacity. This material holds promise for the efficient recovery of Ga(III) from aqueous solutions containing trace amounts of the element.

## Figures and Tables

**Table 1:** Kinetic parameters for the adsorption of Ga(III) onto XAD7-Va.

Kinetic Model	Parameters	Temperature (K)			
		298	308	318	328
Pseudo-first-order	*k*_1_ (1/min)	0.04 ± 0.01	0.05 ± 0.01	0.06 ± 0.01	0.07 ± 0.02
	*q*_*e*,*calc*_ (mg/g)	2.31 ± 0.42	2.34 ± 0.38	2.33 ± 0.35	2.33 ± 0.27
	*R* ^2^	0.9963	0.9959	0.9960	0.9947
	*χ* ^2^	0.004	0.003	0.003	0.003
	*R* ^2^ *_adj_*	0.9937	0.9952	0.9951	0.9956
Pseudo-second-order	*k*_2_ (1/min)	0.02 ± 0.01	0.02 ± 0.01	0.03 ± 0.01	0.04 ± 0.01
	*q*_*e*,*calc*_ (g/mg·min)	2.71 ± 0.46	2.71 ± 0.35	2.63 ± 0.39	2.58 ± 0.49
	*R* ^2^	0.9826	0.9804	0.9806	0.9794
	*χ* ^2^	0.014	0.017	0.017	0.018
	*R* ^2^ *_adj_*	0.9791	0.9765	0.9767	0.9753
Elovich	*a* (g/mg)	1.83 ± 0.26	1.95 ± 0.35	2.35 ± 0.37	2.76 ± 0.41
	*b* (mg/g·min)	0.40 ± 0.08	0.54 ± 0.14	1.20 ± 0.24	2.75 ± 0.47
	*R* ^2^	0.9631	0.9595	0.9602	0.9601
	*χ* ^2^	0.031	0.035	0.035	0.035
	*R* ^2^ *_adj_*	0.9558	0.9514	0.9522	0.9522
Avrami	*k_AV_* (1/min)	0.26 ± 0.04	0.27 ± 0.05	0.30 ± 0.07	0.32 ± 0.09
	*q_AV_* (mg/g)	2.27 ± 0.35	2.31 ± 0.43	2.29 ± 0.29	2.28 ± 0.34
	*n_AV_*	0.18 ± 0.03	0.19 ± 0.04	0.21 ± 0.04	0.22 ± 0.03
	*R* ^2^	0.9958	0.9954	0.9952	0.9938
	*χ* ^2^	0.005	0.004	0.004	0.003
	*R* ^2^ *_adj_*	0.9921	0.9940	0.9939	0.9945

**Table 2:** Thermodynamic parameters for adsorption of Ga(III) onto XAD7-Va.

Δ*H*^0^ (kJ/mol)	Δ*S*^0^ (J/mol·K)	Δ*G*^0^ (kJ/mol)			
		298 K	308 K	318 K	328 K
35.8	137.2	−40.8	−42.2	−43.5	−44.9

**Table 3:** Parameters of tested isotherm model for adsorption of Ga(III) onto XAD7-Va.

Isotherm Model	Parameters	Value
Langmuir	*K_L_* (L mg^−1^)	0.14 ± 0.02
	*q_max_* (mg g^−1^)	28.86 ± 2.35
	*R* ^2^	0.9881
	*χ* ^2^	1.31
	*R* ^2^ *_adj_*	0.9872
Freundlich	*K_f_* (mg g^−1^)	5.36 ± 0.87
	1/*n*	2.38 ± 0.47
	*R* ^2^	0.9417
	*χ* ^2^	6.43
	*R* ^2^ *_adj_*	0.9376
Temkin	*K_T_* (L mg^−1^)	88.33 ± 5.41
	*b* (kJ g^−1^)	1101 ± 294
	*R* ^2^	0.6671
	*χ* ^2^	36.87
	*R* ^2^ *_adj_*	0.6426
Sips	*Q_sat_* (mg g^−1^)	28.79 ± 3.74
	*K_S_* (L mg^−1^)	0.14 ± 0.03
	*n*	1.01 ± 0.12
	*R* ^2^	0.9881
	*χ* ^2^	1.41
	*R* ^2^ *_adj_*	0.9863

**Table 4:** Comparison of adsorption capacities among various materials employed for Ga(III) adsorption.

Adsorbent	Adsorption Capacity (mg/g)	Adsorption Conditions	Reference
Fly ash	2.89	pH = 8.35; t = 24 h; T = 323 K	[36]
Zeolite HY	7.90	pH = 0.5; t = 24 h; T = 293 K	[37]
Unmodified coir	13.75	pH > 2.5; t = 0.5 h; T = 298 K	[38]
Activated carbon	16.00	t = 20 h; T = 298 K	[20]
Oxidized coir	19.42	t = 0.5 h; T = 298 K	[38]
XAD7-Va	28.86	pH > 9; t = 1.5 h; T = 318 K	This paper

**Table 5:** Controllable factors employed in Taguchi design and their levels.

Factor	Level 1	Level 2	Level 3	Level 4
pH	1	4	7	10
Time (min)	15	45	90	120
Initial Ga(III) concentration (mg/L)	1	20	60	110
Temperature (K)	298	308	318	328

**Table 6:** The experimental results and the corresponding S/N ratios for each trial.

pH	Time	Initial Ga(III) Concentration	Temperature	Removal Efficiency	S/N Ratio
1	15	1	298	71.27	37.05
1	45	20	308	85.01	38.59
1	90	60	318	86.95	38.78
1	120	110	328	54.52	34.73
4	15	20	318	75.08	37.51
4	45	1	328	85.71	38.66
4	90	110	298	71.52	37.08
4	120	60	308	88.18	38.90
7	15	60	328	79.42	37.99
7	45	110	318	72.68	37.22
7	90	1	308	86.26	38.71
7	120	20	298	89.38	39.02
10	15	110	308	59.95	35.55
10	45	60	298	87.66	38.85
10	90	20	328	89.72	39.05
10	120	1	318	87.94	38.88
1	15	1	298	71.27	37.05
1	45	20	308	85.01	38.59
1	90	60	318	86.95	38.78
1	120	110	328	54.52	34.73
4	15	20	318	75.08	37.51
4	45	1	328	85.71	38.66

**Table 7:** Response table for signal-to-noise (S/N) ratios (larger is better).

Level	pH	Time	Initial Ga(III) Concentration	Temperature
1	37.29	37.03	38.33	38.01
2	38.04	38.33	38.55	37.94
3	38.24	38.41	38.64	38.10
4	38.09	37.89	36.15	37.61
Delta	0.95	1.38	2.49	0.49
Rank	3	2	1	4
Contribution (%)	9.60	19.89	66.88	3.63

## Data Availability

All the experimental data obtained are presented, in the form of tables and/or figures, in the article.
